# Social activities and long-term depressive-symptoms trajectories among middle-aged and older adults in China: a population-based cohort study

**DOI:** 10.3389/fpsyt.2023.1131084

**Published:** 2023-08-16

**Authors:** Xuhui Lin, Siyue Liu, Zhao Hu, Huilan Xu

**Affiliations:** Department of Social Medicine and Health Management, Xiangya School of Public Health, Central South University, Changsha, Hunan, China

**Keywords:** social activities, the elderly, cohort study, depression symptom, trajectories

## Abstract

**Background:**

The association between social activities and depressive symptoms remains unclear. This study aimed to explore the relationship between social activities at baseline and the long-term depressive-symptoms trajectories among a cohort of middle-aged and older adults in China.

**Methods:**

This study included 13,258 participants aged 45 years and older from the China Health and Retirement Longitudinal Study (CHARLS). Depressive symptoms across four waves from 2011 to 2018 were evaluated using the 10-item Center for Epidemiologic Studies Depression Scale (CESD-10). Four types of social activities were assessed at baseline by self-report: (1) interacting with friends; (2) playing Mahjong, chess, and cards or attending a community club; (3) providing help to family, friends, or neighbors; and (4) attending a sporting or social event or club. Group-based trajectory modeling (GBTM) was used to map depressive-symptoms trajectories during the follow-up period.

**Results:**

Not interacting with friends at baseline was associated with an increased risk of increasing (adjusted odds ratio [aOR]: 1.21, 95% confidence interval [CI]: 1.03, 1.41) and severe-stable (aOR: 1.35, 95% CI: 1.10, 1.65) depressive-symptoms trajectories. Participants who did not play Mahjong, cards, or chess and did not attend a sporting or social event or club at baseline were more likely to have mild-stable, decreasing, increasing, and severe-stable depressive-symptoms trajectories.

**Conclusion:**

Social activities play an important role in long-term depressive-symptoms trajectories in middle-aged and older Chinese adults. Interacting with friends, attending sports, or social clubs may prevent depressive symptoms.

## Introduction

Depression is one of the most common mental disorders and is ranked as the second leading cause of disability worldwide (1); therefore, the study of depressive symptoms as precursors to clinical depression has long been a crucial area of research in both mental and public health. Typically, depressive symptoms are temporary and situation specific. However, they do not always resolve spontaneously without intervention. The key symptoms of depression encompass a range of manifestations, including low mood, loss of appetite, psychomotor slowing, suicidal ideation, sleep disturbance, and feelings of guilt, worthlessness, hopelessness, and helplessness (2).

With the increase in global aging (3), depression in middle-aged and older adults cannot be ignored. A global meta-analysis of depressive symptoms among older adults showed that the prevalence of depression was 35.1% (4). However, a recent meta-analysis conducted in China showed that the pooled overall prevalence of depressive symptoms among older adults was 20.0% (5). It is universally acknowledged that depressive symptoms were associated with adverse outcomes of health. This claim is supported by scholarly evidence that people with depressive symptoms were associated with several chronic diseases such as hypertension, coronary heart disease, diabetes, chronic obstructive pulmonary disease (6), allergic rhinitis, cataracts (7) and chronic kidney disease. Moreover, older adults with depressive symptoms are more likely to have increased healthcare needs and utilization (8), which eventually leads to an augmented burden on health resources (9).

Studies have shown a variety of risk factors associated with depressive symptoms in older adults, such as dissatisfaction with their living situation (10), single adverse life events (11), insufficient sleep (12), poor vision, poor oral health (13) and so on. Few studies have focused on these social factors. For example, research suggests that older adults, especially those who find it difficult to engage in community activities, are associated with experience depressive symptoms (14–16). A cross-sectional study on older adults in Australia also indicated that participating in regular physical activities, such as tennis, dance classes, and vigorous cycling, were associated with decreased depressive symptoms (17). Similarly, encouraging older people with family and friends to establish a broad and diverse social network may associated with decreased depressive symptoms (18).

The social activities of older adults in China can be classified into four categories: sports and fitness, cultural and recreational, interpersonal, and family life-related activities (19). Engaging in pleasant and meaningful social activities can promote coping and recovery from trauma (20). This is because social activities enhance social friendships and meaningful engagement, which can help buffer against stress (21). This can alleviate depressive symptoms. In recent years, a growing body of research has explored different types of activities as coping strategies for different types of stress. A study have shown that older adults who actively participate in social activities obtain sufficient social support, which can help to relieve stress (22). Additionally, social activities were associated with more powerful abilities to cope with negative life events among older adults (22).

However, few studies have explored the relationship between social engagement and long-term depressive-symptoms trajectories among middle-aged and older adults. A trajectory is the process of changing behavior with age or time. A group-based method was used to identify particular groups of individual depressive-symptoms trajectories in the population and to analyze the characteristics of the group members (23). Another study showed that the status of depressive symptoms is heterogeneous from the perspective of long-term trajectories: transient, stable, and fluctuating at variable frequencies (24). Although there have been studies in China based on long-term trajectories analyzing the impact of social engagement on depressive symptoms (25), no previous studies have explored whether the social activities of middle-aged and older adults in China have an impact on long-term depressive-symptoms trajectories.

To fill this research gap, this study aimed to explore the impact of social activities on the long-term depressive-symptoms trajectories among middle-aged and older adults in China. We hope that this study will provide valuable information to help formulate targeted policies and implement multifaceted interventions for the mental health of middle-aged and older adults.

## Materials and methods

### Study population

The study population consisted of participants aged 45 years and above (range: 45–96 years) from the China Health and Retirement Longitudinal Study (CHARLS), an ongoing nationally representative longitudinal study conducted by the National School of Development at Peking University (26). The CHARLS data have been widely used to study health among older adults (27, 28). A total of 17,708 participants were recruited using a multistage probability sampling procedure from 150 counties/districts and 450 communities within 28 provinces at baseline in 2011, with a response rate of 80.5%. Approximately 500 professional interviewers collected the data, and the quality was checked using a computer-assisted personal interviewing system. All participants were followed-up biennially after the baseline survey. In this study, the participants were from four waves of the CHARLS (2011, 2013, 2015, and 2017). CHARLS was approved by the Biomedical Ethics Review Committee of Peking University (IRB00001052-11015), and written informed consent was obtained from all participants.

### Assessment of depressive symptoms

Depressive symptoms across the four waves were evaluated using the 10-item Center for Epidemiologic Studies Depression Scale (CESD-10), given that CESD-10 has shown good validity and reliability in CHARLS (29). The Cronbach’ α coefficient was 0.78–0.79 (30). Participants were asked how often they had experienced any of the ten symptoms listed during the past week. The answers to the 10 items ranged from rarely or never (<1 day), sometimes (1–2 days), occasionally (3–4 days), to most or all of the time (5–7 days). Each item is assigned a score ranging from 0 (rarely) to 3 (most or all of the time). Before summing the item scores, items 5 and 8 were reverse-scored. The total CESD-10 score ranged from 0 to 30, with higher scores indicating greater depressive symptoms. According to a previous study, participants with a total score of 12 or higher are defined as having depressive symptoms (29). Participants with more than two missing values and those without baseline CESD-10 scores were excluded from the study.

### Assessment of social activities

Four types of social activities in the last month at baseline were assessed by self-reporting: (1) interacting with friends; (2) playing Mahjong, chess, or cards; (3) providing help to family, friends, or neighbors who did not pay for the help; and (4) going to a sport, social club, or another kind of club (e.g., dance, Qigong, or community club). The frequency of each type of social activity (almost daily, almost every week, and not regularly) in the previous month was identified and the participants were further classified into three groups: no attendance, seldom (not regularly), and often (combined almost daily and almost every week). Although CHARLS provided information about many other types of social activities, we did not analyze their frequency because of their extremely low prevalence (1.3% for community-related organizations, 0.6% for voluntary or charity work, 0.7% for taking care of a sick or disabled adult, 0.3% for attending an educational or training course, 0.5% for stock investment, and 2.6% for general Internet usage).

### Covariates

Covariates, including sociodemographic information, lifestyle behaviors, and health-related factors, were collected using a structured questionnaire. Sociodemographic information included age, gender, residence location, education, and marital status. Marital status was classified as married or unmarried (separated, divorced, widowed or never married). Education level was assessed by asking participants about their highest level of education completed using a single item and classified as no formal education, primary school, middle or high school, or college or above. Lifestyle behaviors included smoking status (current/former/never), drinking status (current/former/never), sleep duration (<7 h, 7–7.9 h, and ≥8 h), and nap duration (<30 min, 30–59 min, and ≥60 min). Sleep duration was assessed using a single item: “During the past month, how many hours of actual sleep did you average per night? (This may be shorter than the number of hours you spent in bed).” Nap duration was assessed using a single item: “During the past month, what was your average daily nap duration?” Health-related factors included self-rated health, self-reported chronic diseases diagnosed by a physician (hypertension, diabetes, dyslipidemia, chronic lung diseases, chronic kidney disease, heart disease, stroke, arthritis, or rheumatism), history of medication use (antihypertensive, antidiabetic, and lipid-lowering medication), and other functional limitations. Self-rated health was assessed using a single item, “How would you rate your health currently?” Participants were asked to rate their health on a five-point scale with the following response categories: (5) excellent, (4) good, (3) fair, (2) poor, and (1) very poor. They were further classified as good (a combination of 4 and 5), fair, or poor (a combination of 2 and 1). Functional limitations were measured using the Katz Activities of Daily Living (ADL) and Lawton Instrumental Activities of Daily Living (IADL) scales (31, 32). Those who reported needing assistance with any of the six ADLs (dressing, bathing, eating, getting into and out of bed, toileting, and controlling urination and defecation) and/or the five IADLs (preparing a hot meal, shopping for groceries, doing housework, taking medicines, and managing money) were classified as having functional limitations (33).

### Statistical analysis

Data for continuous variables were reported as means and standard deviations (SDs), whereas data for categorical variables were reported as counts and percentages. The depressive-symptoms trajectories were determined using group-based trajectory modeling (GBTM) to map the development of symptoms during the follow-up period. Baseline characteristics were summarized according to the five depressive-symptoms trajectories and differences were compared using chi-square tests and one-way analysis of variance (ANOVA). A censored normal distribution model was created using the Stata *TRAJ* procedure to estimate the mean trajectories of the CESD-10 scores across the four visits (2011, 2013, 2015, and 2017). The depressive-symptoms trajectories of participants with at least two study visits were plotted based on their CESD-10 values. To define the optimal number of depressive-symptoms trajectories, several models were fitted, ranging from a 1-group to a 6-group trajectory model. We selected the model with the best fit based on the following criteria: (1) the average posterior probability of each trajectory group was ≥0.70; (2) the sample size was more than 5.0% of the population; and (3) the minimum absolute value of the Bayesian Information Criterion (BIC). Based on these criteria, a 5-group trajectory model was determined to have the best fit for the data. The association between social activities and probability of belonging to each depressive-symptoms trajectory was evaluated using a multinomial logistic regression model. We calculated odds ratios and 95% confidence intervals (CIs) based on three models: Model 1 adjusted for age and gender. Model 2 adjusted for age, gender, residence location, education, marital status, smoking status, alcohol consumption, sleep duration, and nap duration. Model 3 adjusted for the variables in Model 2 plus self-rated health, self-reported diabetes, hypertension, dyslipidemia, chronic lung disease, chronic kidney disease, heart disease, stroke, and arthritis or rheumatism; use of antihypertensive, antidiabetic, and lipid-lowering medication; and functional limitations. Missing covariate data (10.4%) were imputed using the multiple imputation of chained equations methods, and the pooled results were reported for the five depressive-symptoms trajectories. Sensitivity analyses were conducted as follows: (1) all analyses were repeated using the complete dataset of 12,789 participants without multiple imputations; and (2) gender differences in the association were performed in subgroup analyses, and the interaction was tested using a likelihood ratio test. All analyses were conducted using the Stata software (version 16.0; StataCorp, College Station, TX, USA). Statistical significance was defined as a two-sided *P-*value of <0.05.

## Results

**FIGURE 1:**
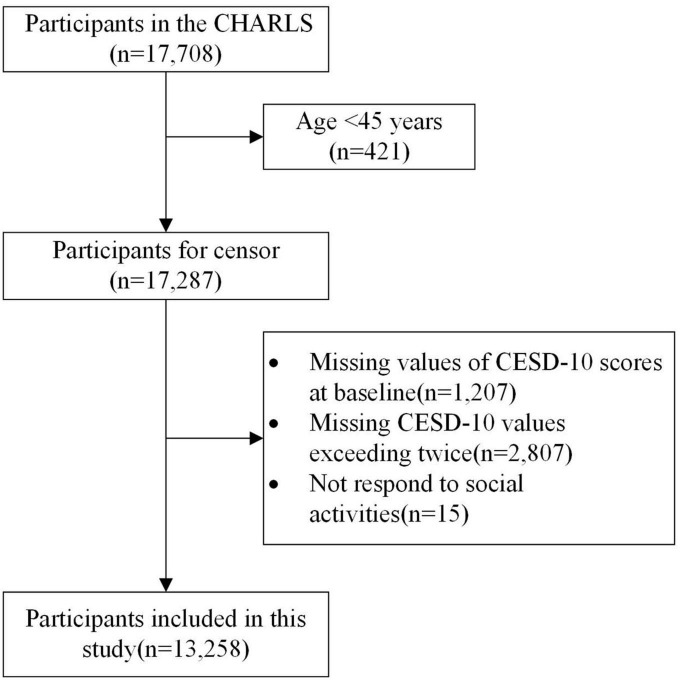
The flow chart of participants included in this study.

Of the 17,708 CHARLS participants at baseline, 421 participants aged younger than 45 years were excluded. A total of 1,207 participants were excluded owing to missing baseline CESD-10 scores. A total of 2,807 participants with more than two missing CESD-10 scores were excluded. Fifteen participants who did not respond to the questions on social activities were also excluded. Finally, 13,258 participants were included in the analysis. A flow chart is shown in Figure 1. The baseline characteristics of the included and excluded participants are shown in Supplementary Table 1.

**FIGURE 2:**
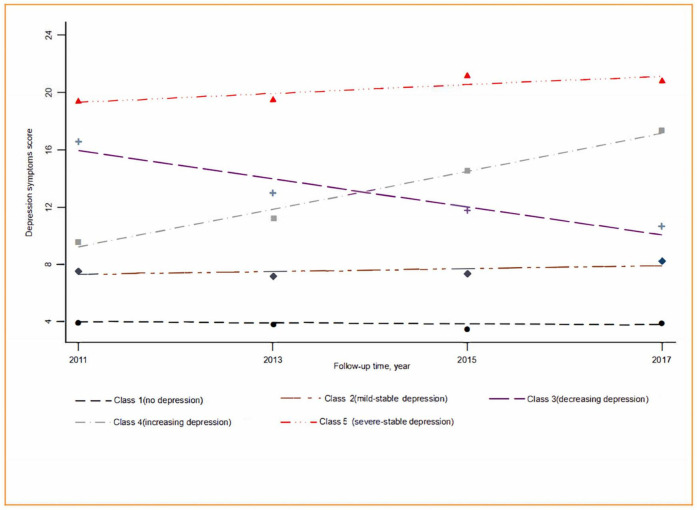
Depressive-symptoms trajectories from 2011 to 2018 among middle-aged and older adults in China.

Five depressive-symptom trajectories were identified in our study using the GBTM analysis (BIC = −135271.05; *n* = 13,258), as shown in Figure 2: no depressive symptoms (Class 1: 31.5%), mild-stable depressive symptoms (Class 2: 41.8%), decreasing depressive symptoms (Class 3: 10.5%), increasing depressive symptoms (Class 4: 10.2%), and severe-stable depressive symptoms (Class 5: 6.0%). The baseline characteristics of the five depressive-symptoms trajectory groups are shown in Table 1. The mean (SD) age was 58.5 (9.0) years, 48.0% of the participants were men, 61.3% lived in rural areas, and 88.8% were married. A total of 3,162 (24.0%) participants had self-reported hypertension, 5.6% had self-reported diabetes, and 1,532 (11.6%) had functional limitations. According to the Chi-square and ANOVA results, there were statistically significant differences between the five groups in terms of age, gender, residence location, education, smoking, alcohol consumption, self-rated health, sleep duration, nap duration; prevalence of hypertension, diabetes, chronic lung disease, chronic kidney disease, heart disease, stroke, arthritis, or rheumatism; and use of antihypertensive, antidiabetic, and lipid-lowering medication (all *P* < 0.05).

**TABLE 1:** Characteristics of participants according to different depressive-symptoms trajectories.

Characteristics	Overall (*n* = 13,258)	No depression (*n* = 4,175)	Mild-stable (*n* = 5,543)	Decreasing (*n* = 1,395)	Increasing (*n* = 1,356)	Severe-stable (*n* = 789)	*P-*value^a^
Age, years	58.5 (9.0)	57.8 (8.9)	58.6 (9.1)	59.9 (9.3)	58.5 (8.8)	59.6 (8.7)	<0.001
Male	6,362 (48.0)	2,446 (58.6)	2,684 (48.4)	532 (38.2)	497 (36.7)	203 (25.7)	<0.001
Rural area	8,124 (61.3)	2,127 (50.9)	3,422 (61.7)	1,001 (71.8)	978 (72.1)	596 (75.5)	<0.001
Married	11,770 (88.8)	3,849 (92.2)	4,940 (89.1)	1,152 (82.6)	1,201 (88.6)	628 (79.6)	<0.001
Education^b^							<0.001
No formal	3,345 (25.2)	714 (17.1)	1,375 (24.8)	486 (34.8)	440 (32.5)	330 (41.8)	
Primary school	5,359 (40.4)	1,483 (35.5)	2,301 (41.5)	612 (43.9)	621 (45.8)	342 (43.3)	
Middle school and above	4,550 (34.3)	1,976 (47.4)	1,866 (33.7)	297 (21.3)	294 (21.7)	117 (14.8)	
Smoking							<0.001
Current	4,105 (31.0)	1,442 (34.5)	1,746 (31.5)	382 (27.4)	374 (27.6)	161 (20.4)	
Formal	1,150 (8.7)	421 (10.1)	483 (8.7)	110 (7.9)	79 (5.8)	57 (7.2)	
Never	8,003 (60.4)	2,312 (55.4)	3,314 (59.8)	903 (64.7)	903 (66.6)	571 (72.4)	
Drinking							<0.001
Current	3,387 (25.5)	1,298 (31.1)	1,424 (25.7)	290 (20.8)	257 (19.0)	118 (15.0)	
Formal	779 (5.9)	228 (5.5)	320 (5.8)	98 (7.0)	89 (6.6)	44 (5.6)	
Never	9,092 (68.6)	2,649 (63.4)	3,799 (68.5)	1,007 (72.2)	1,010 (74.5)	627 (79.5)	
Self-rate health^b^							<0.001
Good	3,186 (24.0)	1,633 (39.1)	1,175 (21.2)	125 (9.0)	215 (15.9)	38 (4.8)	
Fair	6,421 (48.4)	2,068 (49.5)	2,951 (53.2)	545 (39.1)	632 (46.6)	225 (28.5)	
Poor	3,649 (27.5)	472 (11.3)	1,417 (25.6)	725 (52.0)	509 (37.5)	526 (66.7)	
Sleep duration^b^							<0.001
<7 h	6,657 (50.4)	1,534 (36.7)	2,850 (51.6)	931 (67.1)	768 (57.0)	574 (73.4)	
7–7.9 h	2,642 (20.0)	1,091 (26.1)	1,074 (19.5)	180 (13.0)	218 (16.2)	79 (10.1)	
≥8 h	3,901 (29.6)	1,539 (36.9)	1,595 (28.9)	277 (20.0)	361 (26.8)	129 (16.5)	
Nap duration ^b^							<0.001
<30 min	6,421 (48.5)	1,829 (43.8)	2,684 (48.5)	748 (53.7)	699 (51.6)	461 (58.7)	
30–59 min	1,193 (9.0)	389 (9.3)	509 (9.2)	112 (8.0)	110 (8.1)	73 (9.3)	
≥60 min	5,624 (42.5)	1,951 (46.7)	2,341 (42.3)	534 (38.3)	546 (40.3)	252 (32.1)	
Hypertension^b^	3,162 (24.0)	886 (21.2)	1,342 (24.2)	388 (27.8)	347 (25.6)	199 (25.2)	<0.001
Diabetes^b^	738 (5.6)	193 (4.6)	314 (5.7)	96 (6.9)	79 (5.8)	56 (7.1)	0.004
Dyslipidemia^b^	1,234 (9.5)	390 (9.3)	498 (9.0)	140 (10.0)	118 (8.7)	88 (11.2)	0.238
Chronic lung diseases^b^	1,309 (9.9)	265 (6.3)	522 (9.4)	220 (15.8)	159 (11.7)	143 (18.1)	<0.001
CKD^b^	844 (6.4)	153 (3.7)	331 (6.0)	146 (10.5)	100 (7.4)	114 (14.4)	<0.001
Heart disease^b^	1,594 (12.1)	357 (8.6)	607 (11.0)	262 (18.8)	193 (14.2)	175 (22.2)	<0.001
Stroke^b^	257 (1.9)	56 (1.3)	92 (1.7)	48 (3.4)	30 (2.2)	31 (3.9)	<0.001
AR^b^	4,490 (33.9)	901 (21.6)	1,867 (33.7)	715 (51.3)	560 (41.3)	447 (56.7)	<0.001
History of medication^b^							
Antidiabetic	517 (3.9)	142 (3.4)	216 (3.9)	71 (5.1)	52 (3.8)	36 (4.6)	<0.001
Antihypertension	2,500 (18.9)	703 (16.8)	1,064 (19.2)	310 (22.2)	263 (19.4)	160 (20.3)	<0.001
Lipid-lowering	702 (5.4)	198 (4.7)	281 (5.1)	90 (6.5)	73 (5.4)	60 (7.6)	0.004
Functional limitation	1,532 (11.6)	204 (4.9)	554 (1.0)	344 (24.7)	201 (14.8)	229 (29.0)	<0.001

Data are described as mean (SD) or n(%). CKD, chronic kidney disease; AR, arthritis or rheumatism.

^a^*P*-value was determined using the Chi-square test or one-way analysis of variance.

^b^Missing values: 4 for education, 2 for self-rated health, 58 for sleep duration, 20 for nap duration, 66 for hypertension, 116 for diabetes, 235 for dyslipidemia, 50 for chronic lung disease, 80 for CKD, 67 for heart disease, 27 for stroke, 23 for AR, 66 for use of antihypertensive medication, 116 for use of antidiabetic medication, and 235 for use of lipid-lowering medication.

Among the 13,258 CHARLS participants, 1,397 (10.5%) interacted with friends less than once a week, whereas 3,380 (25.5%) reported that they interacted with friends once a week or more. A total of 1,490 (11.2%) individuals reported playing Mahjong, cards, or chess once a week or more, while 281 (2.1%) and 737 (5.6%) participants provided help to family, friends, neighbors, or went to sports or social clubs, respectively. The association between social activities and the depressive-symptoms trajectory after adjusting for age and gender is shown in Supplementary Table 2. The associations between social activities and depressive-symptoms trajectory, adjusted for age, gender, residence location, education, marital status, smoking, drinking, sleep, and nap duration are shown in Supplementary Table 3. The associations between social activities and depressive-symptoms trajectory, adjusted for potential confounders are shown in Table 2. The model 3 has highest pseudo R^2^ value compare to model 1 and 2. Compared to the participants without depressive symptoms who often interacted with friends, the odds ratios of participants were 1.21 (95% CI: 1.03, 1.41) and 1.35 (95% CI: 1.10, 1.65), which correspond to a 21% increase in the odds of being in the increasing depression group and a 35% increase in the odds of being in severe-stable group, respectively. Participants who did not play Mahjong, cards, or chess and did not go to a sport or social club were more likely to belong to the mild-stable, decreasing, increasing, and severe-stable depressive-symptoms trajectory groups. Similar results were obtained after complete data analyses were conducted (Supplementary Table 4).

**TABLE 2:** Association between social activities at baseline and depressive-symptoms trajectory.

Social activities	OR (95% CI)^a^			
	Mild-stable	Increasing	Decreasing	Severe-stable
**Interact with friend**				
Often (*n* = 3,380)	1.00	1.00	1.00	1.00
Seldom (*n* = 1,397)	0.99 (0.85, 1.16)	1.15 (0.90, 1.46)	1.04 (0.82, 1.31)	1.13 (0.83, 1.55)
Not attend (*n* = 8,481)	1.06 (0.96,1.17)	1.21 (1.03, 1.41)	1.09 (0.95,1.26)	1.35 (1.10, 1.65)
**Played Mahjong, chess and cards**				
Often (*n* = 1,490)	1.00	1.00	1.00	1.00
Seldom (*n* = 1,050)	1.10 (0.92, 1.33)	1.64 (1.15, 2.35)	1.47 (1.09, 1.99)	1.68 (1.01, 2.79)
Not attend (*n* = 10,718)	1.37 (1.21, 1.56)	2.45 (1.88, 3.20)	1.50 (1.20, 1.87)	2.72 (1.87, 3.95)
**Provided help to family or friend**				
Often (*n* = 281)	1.00	1.00	1.00	1.00
Seldom (*n* = 695)	1.06 (0.89, 1.27)	0.72 (0.43, 1.19)	0.73 (0.43, 1.22)	0.76 (0.38, 1.51)
Not attend (*n* = 12,282)	1.22 (1.05, 1.42)	0.68 (0.45, 1.05)	0.87 (0.70, 1.08)	0.76 (0.44, 1.33)
**Went to a sport or social club**				
Often (*n* = 737)	1.00	1.00	1.00	1.00
Seldom (*n* = 133)	1.10 (0.72, 1.68)	1.61 (0.69, 3.72)	1.10 (0.48, 2.50)	0.93 (0.47, 1.83)
Not attend (*n* = 12,388)	1.51 (1.26, 1.80)	2.33 (1.57, 3.47)	1.67 (1.18, 2.37)	2.09 (1.22, 3.58)

^a^Adjusted for age, gender, residence location, education, marital status, smoking, drinking, sleep duration, nap duration, self-rated health, self-reported diabetes, hypertension, dyslipidemia, chronic lung diseases, chronic kidney disease, heart disease, stroke and arthritis or rheumatism; use of antihypertensive, antidiabetic, or lipid-lowering medication; and functional limitations. Model fit: χ^2^ = 3209.99; *P* < 0.001; pseudo *R*^2^ = 0.238.

**FIGURE 3:**
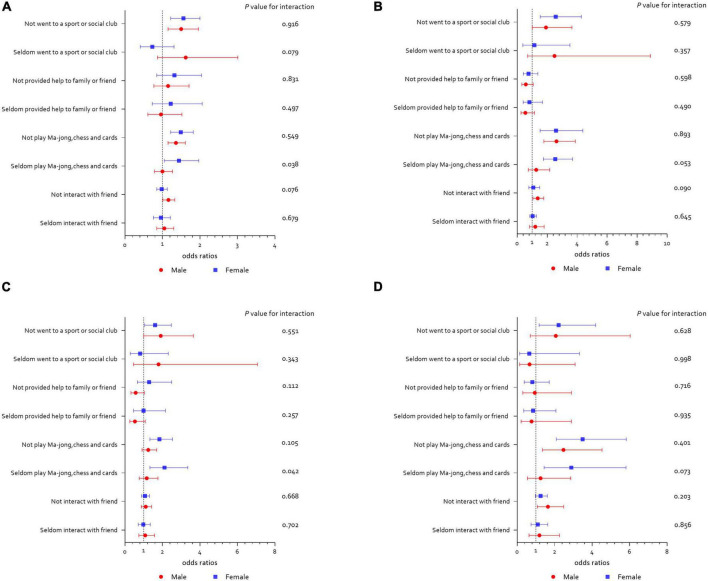
Social activities and long-term depressive-symptoms trajectories according to gender. **(A)** Social activities and mild-stable depressive symptoms; **(B)** social activities and increasing depressive symptoms; **(C)** social activities and decreasing depressive symptoms; **(D)** social activities and severe-stable depressive symptoms.

The association between playing Mahjong, cards, or chess and depressive-symptoms trajectory was more pronounced among woman participants. Compared to men, women who seldom play Mahjong, cards, or chess have higher risk of belonging to the mild-stable (1.44 vs. 1.00, *P* for interaction = 0.038) and decreasing (2.11 vs. 1.16, *P* for interaction = 0.042) depressive-symptoms trajectories. The results are shown in Figure 3.

## Discussion

This study was a population-based investigation of the impact of social activities on long-term depressive-symptoms trajectories in a nationally representative sample of middle-aged and older Chinese adults. Five depressive-symptoms trajectories were identified: no depressive symptoms, mild-stable depressive symptoms, decreasing depressive symptoms, increasing depressive symptoms, and severe-stable depressive symptoms. Participants who did not interact with friends were at a higher risk of belonging to the increasing and severe-stable depressive-symptom groups. Moreover, those who did not play Mahjong, chess, or cards or did not go to a sports or social club were likely to experience different levels of depressive symptoms over time. Women who seldom played Mahjong, chess, and cards were more likely than men to belong to the mild-stable and decreasing depressive-symptoms trajectory groups.

Interactions with friends play an important role in reducing depressive symptoms. Our findings indicated that older adults who did not interact with friends maintained a high or increasing level of depressive symptoms over time, which is consistent with the findings of previous studies (34–36). However, the results of this study differed unexpectedly from those of a similar study conducted in Hong Kong (37). The Hong Kong study found that individuals who had social networks exclusively made up of friends reported higher levels of depression. There could be various reasons for this finding, including the use of different methods to assess various aspects of social interaction among friends. Furthermore, the Hong Kong study’s context suggests that older adults may have relied more on the social support provided by their family members than their friends, which could explain why having only friends in their social network was associated with higher depression levels.

Another finding is that older adults who did not provide help to family or friends were more likely to belong to the long-term trajectory group with mild-stable depressive symptoms, which did not deteriorate over time. A study on this activity stated that although providing appropriate informal help to others can relieve depression, excessive informal help will lead to heavier obligations and fatigue, which can cause depressive symptoms (38). Thus, we may have obtained the results because our study only considered the frequency of social activities and not their duration. Another reason that the relationship between social activity and reduction in depressive symptoms diminishes might be that helping others does not happen in isolation and must be considered together with other social support measures (39).

We found that middle-aged and older adults who did not play Mahjong, chess, or cards tended to be in the severe-stable or increasing depressive-symptoms groups over long-term trajectories, consistent with reports from a previous study that showed that a lack of engagement in these activities can aggravate depressive-symptoms (40). As a popular pastime, playing Mahjong, chess, or cards can increase opportunities for social support so that older adults are not affected by depression (41). There could be a cultural explanation for the fact that some older adults who do not play Mahjong, chess, and cards are in mild-stable or decreasing depressive-symptoms groups. The benefits of establishing social networks by playing games may be diminished owing to the increased anxiety caused by the uncertainty of winning/losing in older people (42). Compared to older men who seldom played, older women who seldom played seem to had a higher risk of developing depressive symptoms. They were also more likely to have fewer opportunities to make close friends on these social networks to prevent depression (43). However, the older women who seldom played Mahjong, chess, or cards were also at higher risk of mild-stable or decreasing depressive symptoms perhaps due to the fact that women were able to better regulate their emotion (44). This means that they can handle negative emotions and pressure better, such as not arguing with others on the poker table. These conflicting results suggest that the complicated relationship between playing Mahjong, chess, or cards and the long-term trajectories of older adults with depression should be further examined in the future.

With regard to sports or social clubs, the findings were similar to those of previous studies (45–47), in that participants who did not go to a sports or social club were more likely to be part of the severe-stable or increasing depressive-symptom groups. This might be because these gatherings create opportunities for them to build their social networks and enjoy a sense of belonging, which can ward off the symptoms of depression (48). Those in the mild-stable or decreasing depressive-symptom groups were mostly those who did not attend sports or social clubs, especially those who engaged in labor work in rural areas. This is probably because they have no free time or energy to join a club after completing their day job (42).

Interacting with friends, playing Mahjong or attending social clubs are elective social activities, which are associated with depression symptoms possibly through two main mechanisms. First, social activities offer the opportunity for social support. And there is broad evidence that social support has positive effects on wellbeing (49, 50). Second, during social activities, no further demands are put on resources needed during typical task-accomplishing processes. As a consequence, recovery processes can take place, which contribute independently to an individual’s wellbeing (51). However, those who are regularly engaged in providing help to friends and family, perhaps they are recipients of help in the past and therefore have some form of social debt to pay back, which are associated with depression symptoms (52).

This study has two novel findings. First, it is the first study of its kind to investigate the link between playing traditional Chinese games, such as Mahjong, chess, and cards, and long-term depressive-symptoms trajectories among middle-aged and older adults in China. Second, the study revealed that the frequency with which older individuals engage in different social activities over an extended period is associated with the extent of changes in their depressive symptoms. This study has several limitations. First, a causal relationship could not be inferred due to the study design. Second, it only explored the impact of four types of social activities on depressive symptoms, and not all social activities were considered. However, the inverse impact of depressive symptoms on these activities has not yet been discussed. Further studies are required to clarify this relationship. Third, the frequency of social activities was explored but not their duration and intensity. Future studies should address the complexity of social activities to explore this relationship. Fourth, the data were self-reported, and information on social activities was collected over the past month. Therefore, recall and measurement biases were unavoidable. Finally, this study did not include variables about the built environment in the analysis. The built environment of older adults may influence social activities, and this may have an impact on the long-term depressive-symptoms trajectory in community-dwelling older adults.

## Conclusion

The main finding of this study is that social activities–mainly interacting with friends, playing Mahjong, chess, or cards, providing help to family and friends, and going to a sports or social club–are related to the long-term depressive-symptoms trajectory among middle-aged and older adults in China. The results of this study indicate that it is necessary to pay attention to the social activities of older adults, which have important implications for effective intervention of depressive symptoms among middle-aged and older adults.

## Data availability statement

The original contributions presented in this study are included in the article/Supplementary material, further inquiries can be directed to the corresponding author. CHARLS data are available at http://charls.pku.edu.cn/pages/data/111/zh-cn.html.

## Ethics statement

The studies involving human participants were reviewed and approved by the Biomedical Ethics Review Committee of Peking University (IRB 00001052-11015 and IRB 00001052-11014). The patients/participants provided their written informed consent to participate in this study.

## Author contributions

HX designed the study. XL and SL wrote and revised the manuscript. ZH conducted the analyses. All authors reviewed the manuscript and approved the submitted version.
