# Supplementary material for: Social activities and long-term depressive-symptoms trajectories among middle-aged and older adults in China: a population-based cohort study
Source: Front Psychiatry. 2023 Aug 16;14:1131084. doi: 10.3389/fpsyt.2023.1131084 (PMC10469621; doi:10.3389/fpsyt.2023.1131084)
Supplement: Supplementary file 1 [file Table_1.docx]

Table S1 Characteristics of included and not included participants at baseline

| Characteristics | Included | Not included | *P*-value ^a^ |
| --- | --- | --- | --- |
| Age, years | 58.5(9.0) | 60.7(12.8) | <0.001 |
| Male | 6362(48.0) | 2109(47.4) | 0.182 |
| Rural area | 8124(61.3) | 2413(54.3) | <0.001 |
| Married | 11770(88.8) | 3647(82.6) | <0.001 |
| Education |  |  | <0.001 |
| No formal | 3345(25.2) | 1458(33.1) |  |
| Primary school | 5359(40.4) | 1593(36.2) |  |
| Middle school and above | 4550(34.3) | 1348(30.7) |  |
| Smoking |  |  | <0.001 |
| Current | 4105(31.0) | 766(17.2) |  |
| Formal | 1150(8.7) | 290(6.5) |  |
| Never | 8003(60.4) | 3391(76.3) |  |
| Drinking |  |  | <0.001 |
| Current | 3387(25.5) | 995(22.4) |  |
| Formal | 779(5.9) | 265(6.0) |  |
| Never | 9092(68.6) | 3187(71.7) |  |
| Self-rate health |  |  | <0.001 |
| Good | 3186(24.0) | 1014(22.8) |  |
| Fair | 6421(48.4) | 1748(39.3) |  |
| Poor | 3649(27.5) | 1557(35.0) |  |
| Sleep duration |  |  | <0.001 |
| <7 h | 6657(50.4) | 1447(50.1) |  |
| 7-7.9h | 2642(20.0) | 494(17.1) |  |
| ≥8h | 3901(29.6) | 948(32.8) |  |
| Nap duration |  |  | 0.793 |
| <30 min | 6421(48.5) | 1445(49.1) |  |
| 30-59 min | 1193(9.0) | 256(8.7) |  |
| ≥60 min | 5624(42.5) | 1244(42.2) |  |
| History of chronic disease |  |  |  |
| Hypertension | 3162(24.0) | 1122(26.2) | 0.003 |
| Diabetes | 738(5.6) | 255(6.0) | 0.372 |
| Dyslipidemia | 1234(9.5) | 361(8.6) | 0.097 |
| Chronic lung diseases | 1309(9.9) | 472(10.6) | 0.155 |
| Chronic kidney disease | 844(6.4) | 262(6.1) | 0.481 |
| Heart disease | 1594(12.1) | 499(11.6) | 0.405 |
| Stroke | 257(1.9) | 156(3.6) | <0.001 |
| Arthritis or rheumatism | 4490(33.9) | 1283(29.7) | <0.001 |
| History of medication |  |  |  |
| Antidiabetic | 517(3.9) | 195(4.4) | 0.051 |
| Antihypertension | 2500(18.9) | 912(20.5) | 0.081 |
| Lipid-lowering | 702(5.3) | 209(4.7) | 0.870 |
| Functional limitation | 1532(11.6) | 1023(23.0) | <0.001 |

Data were presented as mean(SD) or n(%).

^a^ P-value was determined by the Chi-square test or student’s t test

TableS2 Association between social activities and trajectories of depression symptoms in model 1

| Social activities | OR(95%CI)^a^ | | | |
| --- | --- | --- | --- | --- |
|  | Mild-stable | Increasing | Decreasing | Severe-stable |
| Interact with friend |  |  |  |  |
| Often | 1.00 | 1.00 | 1.00 | 1.00 |
| Seldom | 1.02(0.88,1.18) | 1.26(1.01,1.58) | 1.08(0.86,1.36) | 1.30(0.98,1.74) |
| Not attend | 1.09(0.99,1.19) | 1.27(1.10,1.47) | 1.11(0.96,1.28) | 1.42(1.18,1.72) |
| Played Ma-jong, chess and cards |  |  |  |  |
| Often | 1.00 | 1.00 | 1.00 | 1.00 |
| Seldom | 1.21(1.02,1.45) | 2.00(1.43,2.82) | 1.77(1.32,2.37) | 2.10(1.30,3.40) |
| Not attend | 1.56(1.38,1.76) | 3.26(2.55,4.18) | 1.94(1.57,2.39) | 3.88(2.73,5.51) |
| Provided help to family or friend |  |  |  |  |
| Often | 1.00 | 1.00 | 1.00 | 1.00 |
| Seldom | 1.16(0.84,1.61) | 0.84(0.53,1.35) | 0.84(0.51,1.38) | 0.89(0.48,1.67) |
| Not attend | 1.37(1.03,1.82) | 0.87(0.59,1.29) | 1.05(0.69,1.59) | 1.01(0.60,1.71) |
| Went to a sport or social club |  |  |  |  |
| Often | 1.00 | 1.00 | 1.00 | 1.00 |
| Seldom | 1.13(0.75,1.69) | 1.74(0.79,3.84) | 1.16(0.52,2.59) | 1.09(0.31,3.85) |
| Not attend | 2.15(1.82,2.53) | 4.87(3.37,7.04) | 3.51(2.53,4.87) | 5.37(3.27,8.81) |

^a^ adjusted for age and gender;

Model fit: χ^2^=1172.81;*P*<0.001; pseudo *R*^2^=0.094

Table S3 Association between social activities and trajectories of depression symptoms in model 2

| Social activities | OR(95%CI)^a^ | | | |
| --- | --- | --- | --- | --- |
|  | Mild-stable | Increasing | Decreasing | Severe-stable |
| Interact with friend |  |  |  |  |
| Often | 1.00 | 1.00 | 1.00 | 1.00 |
| Seldom | 1.04(0.96,1.12) | 1.29(1.02,1.62) | 1.12(0.90,1.39) | 1.31(0.98,1.77) |
| Not attend | 1.10(1.05,1.16) | 1.31(1.13,1.52) | 1.15(1.01,1.32) | 1.48(1.22,1.79) |
| Played Ma-jong, chess and cards |  |  |  |  |
| Often | 1.00 | 1.00 | 1.00 | 1.00 |
| Seldom | 1.14(0.96,1.37) | 1.78(1.26,2.52) | 1.56(1.16,2.10) | 1.86(1.14,3.01) |
| Not attend | 1.45(1.28,1.64) | 2.78(2.16,3.58) | 1.63(1.62,1.64) | 3.13(2.19,4.48) |
| Provided help to family or friend |  |  |  |  |
| Often | 1.00 | 1.00 | 1.00 | 1.00 |
| Seldom | 1.10(0.81,1.50) | 0.79(0.49,1.28) | 0.77(0.46,1.27) | 0.84(0.44,1.60) |
| Not attend | 1.30(1.12,1.50) | 0.79(0.53,1.19) | 0.95(0.64,1.42) | 0.90(0.52,1.54) |
| Went to a sport or social club |  |  |  |  |
| Often | 1.00 | 1.00 | 1.00 | 1.00 |
| Seldom | 1.06(0.70,1.59) | 1.49(0.67,3.33) | 1.03(0.46,2.31) | 0.88(0.24,3.18) |
| Not attend | 1.62(1.37,1.90) | 2.65(1.81,3.88) | 1.84(1.31,2.59) | 2.39(1.45,3.93) |

^a^ adjusted for age, gender, living residence, education, marital status, smoking status, drinking status, sleep duration and nap duration;

Model fit: χ^2^=1620.00;*P*<0.001; pseudo *R*^2^=0.127

Table S4 Association between social activities and trajectories of depression symptoms in complete data

| Social activities | OR(95%CI)^a^ | | | |
| --- | --- | --- | --- | --- |
|  | Mild-stable | Increasing | Decreasing | Severe-stable |
| Interact with friend |  |  |  |  |
| Often | 1.00 | 1.00 | 1.00 | 1.00 |
| Seldom | 1.00(0.86,1.17) | 1.13(0.88,1.45) | 1.02(0.80,1.30) | 1.14(0.82,1.58) |
| Not attend | 1.07(0.97,1.18) | 1.17(1.00,1.37) | 1.10(0.95,1.29) | 1.38(1.12,1.70) |
| Played Ma-jong, chess and cards |  |  |  |  |
| Often | 1.00 | 1.00 | 1.00 | 1.00 |
| Seldom | 1.13(0.94,1.36) | 1.73(1.19,2.50) | 1.49(1.10,2.03) | 1.84(1.09,3.11) |
| Not attend | 1.40(1.23,1.59) | 2.56(1.95,3.35) | 1.51(1.21,1.88) | 2.96(2.01,4.36) |
| Provided help to family or friend |  |  |  |  |
| Often | 1.00 | 1.00 | 1.00 | 1.00 |
| Seldom | 1.07(0.76,1.50) | 0.66(0.40,1.11) | 0.72(0.42,1.21) | 0.80(0.39,1.65) |
| Not attend | 1.21(0.90,1.63) | 0.65(0.43,1.01) | 0.87(0.56,1.35) | 0.83(0.45,1.53) |
| Went to a sport or social club |  |  |  |  |
| Often | 1.00 | 1.00 | 1.00 | 1.00 |
| Seldom | 1.09(0.71,1.66) | 1.63(0.70,3.79) | 1.11(0.49,2.55) | 0.66(0.14,3.09) |
| Not attend | 1.52(1.27,1.82) | 2.29(1.54,3.41) | 1.68(1.18,2.39) | 2.12(1.24,3.65) |

^a^ adjusted for age, gender, residence location, education, marital status, smoking, drinking, sleep duration, nap duration, self-rated health, self-reported diabetes, hypertension, dyslipidemia, chronic lung diseases, chronic kidney disease, heart disease, stroke and arthritis or rheumatism; use of antihypertensive, antidiabetic, or lipid-lowering medication; and functional limitations.
